# Expert validation of prediction models for a clinical decision-support system in audiology

**DOI:** 10.3389/fneur.2022.960012

**Published:** 2022-08-23

**Authors:** Mareike Buhl, Gülce Akin, Samira Saak, Ulrich Eysholdt, Andreas Radeloff, Birger Kollmeier, Andrea Hildebrandt

**Affiliations:** ^1^Department of Medical Physics and Acoustics, Carl von Ossietzky Universität Oldenburg, Oldenburg, Germany; ^2^Cluster of Excellence Hearing4all, Carl von Ossietzky Universität Oldenburg, Oldenburg, Germany; ^3^Department of Psychological Methods and Statistics, Carl von Ossietzky Universität Oldenburg, Oldenburg, Germany; ^4^Universitätsklinik für Hals-Nasen-Ohren-Heilkunde, Evangelisches Krankenhaus Oldenburg, Oldenburg, Germany; ^5^Hörzentrum Oldenburg gGmbH, Oldenburg, Germany; ^6^Hearing Speech and Audio Technology, Fraunhofer Institute for Digital Media Technology (IDMT), Oldenburg, Germany

**Keywords:** precision audiology, CDSS, expert validation, audiological diagnostics, expert knowledge, machine learning, CAFPAs

## Abstract

For supporting clinical decision-making in audiology, Common Audiological Functional Parameters (CAFPAs) were suggested as an interpretable intermediate representation of audiological information taken from various diagnostic sources within a clinical decision-support system (CDSS). Ten different CAFPAs were proposed to represent specific functional aspects of the human auditory system, namely hearing threshold, supra-threshold deficits, binaural hearing, neural processing, cognitive abilities, and a socio-economic component. CAFPAs were established as a viable basis for deriving audiological findings and treatment recommendations, and it has been demonstrated that model-predicted CAFPAs, with machine learning models trained on expert-labeled patient cases, are sufficiently accurate to be included in a CDSS, but it requires further validation by experts. The present study aimed to validate model-predicted CAFPAs based on previously unlabeled cases from the same data set. Here, we ask to which extent domain experts agree with the model-predicted CAFPAs and whether potential disagreement can be understood in terms of patient characteristics. To these aims, an expert survey was designed and applied to two highly-experienced audiology specialists. They were asked to evaluate model-predicted CAFPAs and estimate audiological findings of the given audiological information about the patients that they were presented with simultaneously. The results revealed strong relative agreement between the two experts and importantly between experts and the prediction for all CAFPAs, except for the neural processing and binaural hearing-related ones. It turned out, however, that experts tend to score CAFPAs in a larger value range, but, on average, across patients with smaller scores as compared with the machine learning models. For the hearing threshold-associated CAFPA in frequencies smaller than 0.75 kHz and the cognitive CAFPA, not only the relative agreement but also the absolute agreement between machine and experts was very high. For those CAFPAs with an average difference between the model- and expert-estimated values, patient characteristics were predictive of the disagreement. The findings are discussed in terms of how they can help toward further improvement of model-predicted CAFPAs to be incorporated in a CDSS for audiology.

## Introduction

Audiological diagnostics mostly relies on test batteries of audiological measures conducted on a patient in need. Experts in audiology characterize patients' hearing impairment by combining the knowledge derived from those audiological measures and additional information from anamnesis as well as their subjective impression of the respective patient. However, experts' experience differs depending on the number of previously treated patients and the range of seen cases (1). On the other hand, large amounts of diverse patient data are available in clinical databases which originate from different audiological tests. Thus, theoretically, the knowledge saved in different databases could be made available to any audiologist with different levels of expertise. This is one long-term goal of the current research.

Toward precision audiology, the clinical decision-support system (CDSS) provides the potential to improve the objectivity of audiological diagnostics by supporting experts with information about probabilities for different audiological findings or treatment recommendations, such as the usage of hearing devices (2). Thereby, less experienced professionals could be supported by a CDSS with an expanded basis of diagnostic knowledge. However, more experienced experts could benefit from the statistical knowledge fed into a CDSS, which exploits a large amount of data and derives knowledge about base rates and association patterns between features that are relevant for audiological recommendations (2, 3).

Currently, CDSSs are not widely adopted in audiology. This is due to a couple of challenges to be solved, such as the integration of different data sources for the same audiological finding (4), the integration of CDSS into the clinical decision-making process of experts (5), and the accomplishment of interpretability of algorithms implemented into a CDSS by clinicians (3). To overcome the latter challenge, it has been recommended to develop CDSS in collaboration with domain experts in the respective medical field (6–8). Expert knowledge can be incorporated into the developmental process in different regards: First, when planning a CDSS, concepts and definitions need to be discussed with domain experts (2). Second, highly-experienced experts can be asked to provide insights into their decision-making process or can be asked to gain insights into the decision-making process of a trained algorithm to be implemented in a CDSS (3). Furthermore, domain experts are needed to provide labels, i.e., to estimate audiological findings, if those are not yet available in a certain database (unlabeled data) [e.g., (9, 10)]. Finally, whenever algorithms were trained on an existing database (3, 11), domain experts can be asked to validate machine-predicted labels (10, 12, 13), and the concordance between experts' and algorithmic decisions can be statistically evaluated (9).

In audiology, some CDSS approaches exist for different decision types of the field. For example, a CDSS has been designed for tinnitus diagnosis and therapy (14) and another one for diagnosing idiopathic sudden hearing loss (15), and for the selection of a suitable hearing aid device type (16). However, these approaches do not rely on test batteries containing a combination of audiological measurements to comprehensively characterize patients. For such a purpose, Sanchez-Lopez et al. (17, 18) performed a classification of hearing-impaired patients based on published research data. Their auditory profiles classify patients along the dimensions of audibility- and non-audibility-related distortions. Importantly, their approach combines data-driven knowledge with audiological model assumptions (17).

**Figure 1:**
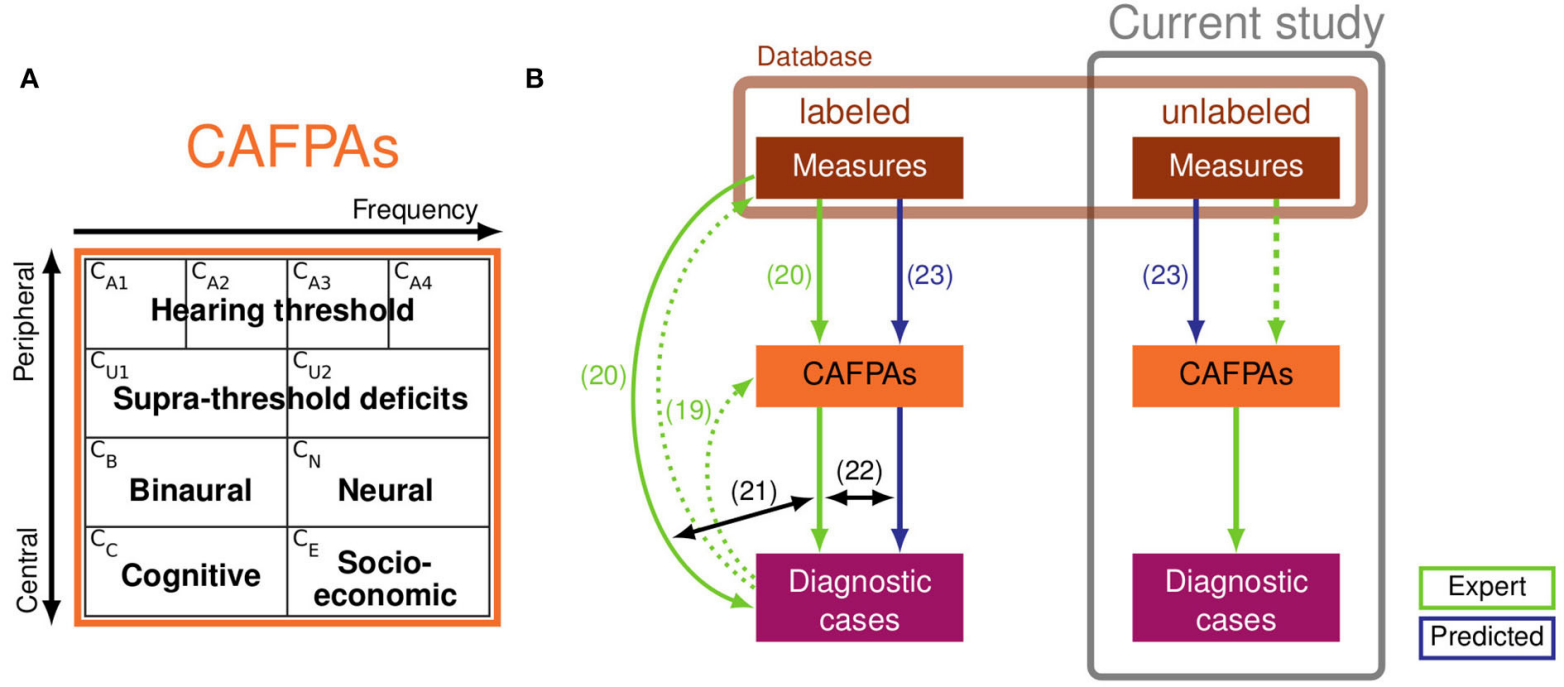
**(A)** Definition of Common Audiological Functional Parameters (CAFPAs). From left to right, the functional aspects CA1-CA4 and CU1-CU2 are frequency-dependent, and from top to bottom, the functional aspects range from peripheral to central. The CAFPAs are defined on a continuum ranging on the interval [0 1], with 0 representing “normal” and 1 representing “maximally impaired”. Panel **(A)** of the figure was taken from Buhl (22). **(B)** Schematic representation of the clinical decision-support system (CDSS) by Buhl (22) (left part, based on labeled data) and relationships to the current study (right, based on unlabeled data). Labeled and unlabeled measurement data originate from the same database (light brown box). Light green arrows depict expert knowledge and blue arrows depict statistical predictions of CAFPAs. Numbered arrows represent contributions of previous studies: collection of expert knowledge in the opposite direction of audiological diagnostics (19); collection of expert knowledge based on individual patients from the currently used database (20); comparison of classification based on audiological measures vs. expert-estimated CAFPAs (21); comparison of classification based on expert-estimated CAFPAs vs. model-predicted CAFPAs. The prediction models were developed by Saak et al. (23) based on the expert-estimated CAFPAs from Buhl et al. (20). The prediction models were derived based on labeled patients (left) and applied to unlabeled patients (right). The experts' task in the current study was to validate the model-predicted CAFPAs (dashed light green arrow) and to estimate audiological findings for unlabeled patients.

Aiming to further ameliorate clinical applicability, Buhl et al. (19–22) and Saak et al. (23) rendered a series of development steps toward a CDSS for audiology, which strongly relies on expert knowledge and is targeted toward future interpretability and integration across different data sources. The CDSS should operate on diverse clinical databases, and it aims at covering the complete audiological decision-making process, including the classification of audiological findings for given patients, as well as suggesting appropriate treatment recommendations (summarized as diagnostic cases). In the proposed CDSS, Common Audiological Functional Parameters (CAFPAs; 19) were employed as an interpretable intermediate layer between audiological tests and diagnostic cases (cf. Figure 1B). CAFPAs were thus introduced as abstract parameters that aim to cover all relevant functional aspects of the human auditory system, while not depending on the exact choice of audiological measures applied to a patient (19). Figure 1A provides an overview of the defined CAFPAs which represent an abstract and common data format based on which different audiological test batteries can be combined and compared, given that a link from a respective measurement to CAFPAs has been established. Buhl et al. (19) introduced the choice of 10 CAFPAs and established the first link to audiological measures and diagnostic cases by means of an expert survey in the inverse direction of the audiological diagnostic process. Thus, 11 audiological experts estimated CAFPAs and distributions of audiological measurement outcomes for given diagnostic cases. This study provided a proof of concept and demonstrated the feasibility of the CAFPA approach.

Aiming to establish a link to individual patients which can be used as training data for machine learning approaches, by means of a second expert survey conducted with 12 experts, Buhl et al. (20) collected CAFPA labels and diagnostic cases for the given measurement outcomes of an existing audiological database. The respective database of individuals with mild-to-moderate hearing impairment contained patients' results on the audiogram, one speech test, and loudness scaling. The audiological measures were visually summarized on result sheets for every patient. The patient data was sorted into categories corresponding to expert-estimated diagnostic cases (labels), and probability density functions were derived for each category and each measurement parameter as well as CAFPA. Thereby, plausible distributions that can be used as training data for classifying diagnostic cases were obtained.

Furthermore, Buhl et al. (21) investigated if CAFPAs provide similar information as included in the audiological measurements and, consequently, if the classification in a CDSS can be performed based on the CAFPAs as intermediate representation instead of directly based on the measurements. For this purpose, classification was performed based on measurements and CAFPAs, employing the training distributions from Buhl et al. (20), including cross-validation. These analyses revealed that, in most cases, approximately the same classification performance in terms of sensitivity and specificity was achieved by CAFPAs as with direct measurements. This means that they contain all the relevant information that is important for classification.

In the above-summarized studies, the relationships between audiological measurements and CAFPAs were established based on expert knowledge only. Thus, the link was not quantified by prediction models and therefore the association pattern could not be used as envisaged in the use case of a CDSS, where CAFPAs for individual, new patients need to be automatically predicted. Aiming to establish an automatic prediction of CAFPAs, Saak et al. (23) statistically derived CAFPAs based on the CAFPA expert labels (collected for 240 out of 595 patients included in the database) and the corresponding outcomes of audiological measures from Buhl et al. (20). This was done by means of regularized regression models (with lasso and elastic net penalties) and random forests. The trained prediction models were shown to have an adequate to good performance, with coefficients of determination (*R*^2^) between 0.6 and 0.7 for the CAFPAs related to the hearing threshold. However, the neural CAFPA CN showed insufficient predictive performance (0.17). As compared with the expert labels, the statistical models tended to predict fewer extreme values for CAFPAs (23). Saak et al. (23) also analyzed the importance of different audiological measures (features) for the prediction and demonstrated that the models indicated audiologically plausible relationships between the measurement outcomes and the CAFPAs. Finally, Saak et al. (23) applied the trained models to predict CAFPAs for the unlabeled part of the database and provided the first consistency check of the model-derived CAFPAs by means of an unsupervised learning approach. More specifically, cluster analysis identified five plausible groups of individuals which were in line with the audiological findings. However, no comparison with “true” labels for audiological findings was possible as expert-estimated diagnostic cases (assumed as ground truth) were not available for the unlabeled patients.

Aiming for further validation of statistically derived CAFPA values, to connect all components, and to finally build a CDSS operable for individual patients (based on labeled data), Buhl (22) applied the classification approach from Buhl et al. (21) to technically evaluate the predictions in the use case of a CDSS (Figure 1B, lower left part). The classification was performed on expert-estimated CAFPAs and model-predicted CAFPAs. It has then been investigated which CAFPAs were relevant for high classification performance in different diagnostic decisions. Furthermore, the interpretability of the system was assessed. It was shown that predicted CAFPAs lead to a similar classification of patients into the different diagnostic cases [prediction accuracy of 0.64–0.78 (depending on the investigated audiological parameter) for optimal weighting of CAFPAs]. The predicted CAFPAs can in general already be used in the classification, but some misclassifications occur that can both be related to the fact that less extreme CAFPAs are predicted by the regression models (23), and to the properties of the data set. However, for a definitive validation of the statistically derived CAFPAs, especially for unlabeled patients, their evaluation by independent experts remains indispensable.

For the purpose of investigating if the current CAFPA prediction can plausibly be applied to unlabeled patients (and consequently to new individual patients in the use case of a CDSS) and to further investigate the properties of the prediction models, the present study aims at an expert validation of the statistically derived CAFPAs [blue and green (dashed) arrows in Figure 1B, right part]. Two highly-experienced audiological experts were asked to assess model-predicted CAFPAs given the measurement outcomes of individual patients and to update the values if they considered a given model-derived CAFPA to be inappropriate. The deviations between model-predicted and expert-validated CAFPAs are statistically analyzed to investigate how disagreements between the model and experts might depend on audiological measurements and to understand how the CAFPA prediction could further be improved. In addition, experts were asked to also estimate audiological findings based on the given measurement data (for the purpose of collecting corresponding labels for diagnostic cases, cf. Figure 1B, lower right part) and to fill out a short questionnaire asking about how they approached the CAFPA evaluation task.

Specifically, the study aimed to provide an answer to the following research questions (RQs):

1. What is the magnitude of relative and absolute agreement of experts with model-predicted CAFPAs? Whereas the relative agreement indicates whether experts and statistical models provide CAFPAs leading to equivalent rank orders of the evaluated patients, the absolute agreement indicates average deviations from the opinion of experts and models across all patients. Both are relevant criteria to understand the overlap between automatic and expertise-based audiological decision-making based on CAFPAs.
2. If a disagreement between model-predicted and expert-validated CAFPAs exists, does it depend on certain characteristics of the patients' test data?
3. Are the estimated audiological findings consistent with expert labels from previous studies collected from patients in the same database?
4. Is the applied expert validation approach a reliable check of the model-predicted CAFPAs?

## Materials and methods

### Data set and audiological experts

For the present study, patients' data displayed to the experts along with model-predicted CAFPAs [as estimated by Saak et al. (23)] were provided by the Hörzentrum Oldenburg gGmbH. The dataset contained *N* = 595 cases for which data were available on medical history, speech recognition in noise performance [Goettingen sentence test, GOESA (24)], two audiological measurements [audiogram and adaptive categorical loudness scaling (25)], and performance on two cognitive tests [German vocabulary test, WST (26); and DemTect (27)]. Patients varied with respect to their degree of hearing loss. A detailed description of the database can be found in Gieseler et al. (28). For *n* = 240 patients, expert labels for CAFPAs and audiological findings were collected by Buhl et al. (20).

The model-predicted CAFPAs for unlabeled patients were taken from Saak et al. (23), where three statistical learning models (lasso regression, elastic net, and random forests) were trained based on 80% of the labeled patients of Buhl et al. (20) and evaluated based on the remaining 20%. The prediction for the 355 existing unlabeled patients was performed using these trained models. Thus, for each statistical learning algorithm, the predictions were obtained by averaging the predicted CAFPAs across 20 models derived from 20 different missing imputed data sets. The code running the prediction models was published along with Saak et al. (23), and it has been applied without any changes. All models performed well, but they were slightly different in their performance accuracy. To account for variation in model performance for the CAFPAs to be evaluated by the experts enrolled in the present study, 50% of the evaluated cases were displayed with estimated CAFPAs based on the best performing model for the respective CAFPA. For the second half of the cases, CAFPAs were taken from the respective worst-performing models.

Two highly-experienced experts (authors AR and UE) evaluated the model-predicted CAFPAs. Both have substantial scientific and clinical experience of more than 20 years (with more than 7,500 seen patients), including all degrees of hearing loss and treatment options. The experts are familiar with the measurements presented in the expert validation survey as well as with measurements performed in clinical practice and their combined interpretation with additional information about patients.

Due to their elaborated experience, two experts were estimated to be sufficient for the purpose of this study. In addition, the experts involved here did not participate in the previous surveys (19, 20) and thereby their expert knowledge was not yet depicted in the current prediction models. This allows for an independent view on the predicted CAFPAs. Moreover, the statistical analysis of differences between the model-predicted and expert-validated CAFPAs (cf. Section Statistical analyses) is better interpretable if the comparison between statistical and expertise-based prediction is performed by individual experts.

### Expert survey design

**Figure 2:**
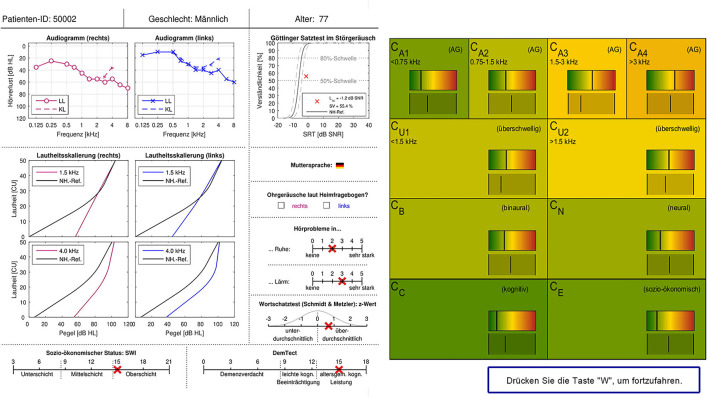
Patient data and CAFPA evaluation sheet as implemented in the electronic version of the expert survey. Patient cases were displayed one at a time. The survey sheet is shown in German as the survey was conducted in Germany. For the main terms, a translation is given in the following. Upper row: Patient ID, gender, and age. Measurements: Audiogram (right and left), LL: air conduction, KL: bone conduction, and hearing loss plotted over frequency. Goettingen sentence test (GOESA) in noise, intelligibility plotted over SRT. Loudness scaling (Adaptive CAtegorical LOudness Scaling (ACALOS); right and left), loudness plotted over level, and black line: normal-hearing reference. Native language. Tinnitus according to the home questionnaire (right and left). Hearing problems in quiet and in noise (scale from none to very much). Verbal intelligence test: z-score (negative scores: below average, positive scores: above average). Socio-economic status: lower class, middle class, and upper class. DemTect: suspicion of dementia, mild cognitive impairment, and age-specific normal cognitive abilities. CAFPAs: the meaning of the different parameters is given in Figure 1A.

The original survey design from Buhl et al. (20) was adopted and implemented as an electronic survey on PsychoPy 3 Builder (29). Same as in Buhl et al. (20), the information sheet of a given patient was presented to the expert on the left side of the screen (see Figure 2), one patient at a time. On the right side of the screen, statistically predicted CAFPAs for the given patient were presented on the range highlighted by the traffic-light color. A visual analog scale of the same range was displayed below. Experts were requested to use this scale and indicate their estimate for all 10 CAFPAs using the respective slider. They were instructed that their slider setting could be perfectly overlapping with the bar indicating the model estimate, or it could deviate from it. The experts were clearly informed about the meaning of the displayed CAFPAs. They thus knew that these were estimates originating from trained statistical algorithms by Saak et al. (23). After placing the slider for all CAFPAs, the experts were able to proceed to the next page by pressing the button displayed at the lower right corner of the screen. On the next page, the same patient's data were displayed again, but on the right side of the screen, audiological findings were now listed, asking the experts to select those that they considered appropriate (multiple answers were allowed). Audiological findings were as follows: 1. normal hearing; 2. cochlear hearing loss (with the options high-frequency, middle-frequency, low-frequency, or broadband hearing loss); 3. conductive hearing loss; 4. central hearing loss. After indicating the appropriate audiological finding(s), experts could proceed with evaluating the next patient. There were separate blocks of 15 patients each, such that experts could interrupt their evaluation for shorter or longer breaks. It was possible to restart the survey on another day and continue with the block of patients who were not yet evaluated before. Experts were not informed about the repeated patients. These were just displayed randomly to them in between new patient cases. Expert 1 evaluated CAFPAs predicted for 150 cases which were randomly selected out of the 355 existing unlabeled patient cases. The cases were chosen to equally correspond to the five clusters of Saak et al. (23) to represent different hearing loss degrees as uniformly as possible. Half of them were predicted with the best and worst performing models, respectively. For evaluating the within-expert agreement, 15 of these cases were presented two times to Expert 1. Expert 2 evaluated 15 patient cases repeatedly, 12 out of those were also evaluated by Expert 1. Expert 2 only received patient cases associated with the CAFPAs predicted by the models with the best performance accuracy.

After each session of 15 cases, a form was displayed, and experts were asked to indicate their confidence in deciding on the CAFPAs' values and the suggested audiological findings. Furthermore, at the end of the survey, they were requested to reveal their expert validation approach and to indicate which measurement information they used while updating each CAFPA. More specifically, we asked whether experts have evaluated the displayed measurements or the statistically estimated CAFPAs first and whether they considered the predicted CAFPAs at all. Furthermore, for each measurement, a list of all CAFPAs was displayed to the experts one by one, and they were asked to mark whether a certain CAFPA was relevant for a given measurement. If none of the CAFPAs was considered to be related to a specific measurement, experts were asked to choose the reason from the options, “The measurement is not known to me,” “The measurement is not important for the characterization of patients,” or “Not possible to decode or represent in CAFPAs.” In addition, the expert's approach to the expert validation task was assessed by a multiple-choice question where different potential approaches or components of those were suggested (Supplementary Tables A1, A2 for details).

### Statistical analyses

All analyses were conducted with the R Software for Statistical Computing (30). To estimate the stability of the CAFPA ratings within and relative agreement across experts, as well as the relative agreement between the model-predicted and expert-validated CAFPA, intraclass correlation coefficients (ICCs) were computed along with their 95% confidence intervals (CIs). The ICC is a widely used tool for measuring inter-rater agreement. It indicates a correlation within the same class of data (here repeated measurements of CAFPAs by different sources: Statistical model, Expert 1, and Expert 2). Whereas the correlation coefficient refers to different variables, the ICC is a correlation of the same variable measured in different conditions. The psych package (31) has been used for this purpose by applying a two-way mixed-effects model [ICC3k (32)]. The relative agreement between experts, as well as between statistical models and experts, indicates whether the raters were ranking the patient cases in terms of CAFPAs in an approximately equivalent order. If the patients' rank orders were approximately overlapping between raters, the ICC would take on a value close to 1. Within-expert stability and cross-expert agreement were taken as necessary preconditions (reliability) for estimating the relative overlap between experts' ratings vs. those of the statistical models.

Not only rank order agreement but also absolute agreement was relevant to understand the overlap between model-predicted and expert-validated CAFPAs. To estimate absolute agreement, a series of linear mixed effect regression (LMER) models were fitted by means of the package lme4 (33), separately for each CAFPA as an outcome variable. The condition model-predicted vs. expert-validated was dummy coded (0 = statistical model). Random intercepts were included when regressing a CAFPA onto the within-patient condition factor to estimate the absolute difference between CAFPA ratings of Expert 1 vs. the statistical models. Given the dummy coded within-patient factor, a negative β-weight (fixed effect) will indicate higher CAFPA values provided by the statistical models on average across patients as compared with the expert. In analogy, a positive β-weight indicates the expert to rate a certain CAFPA higher than the model. These analyses were only based on data from Expert 1, because Expert 2 evaluated only a few patients, but repeatedly multiple times. Per design, the data from Expert 2 were collected for reliability estimates with many repetitions.

Last, we aim to test whether the measured audiological data of the patients can explain potentially observed differences between the model-predicted and expert-validated CAFPAs. Thus, patients' audiological measures were included as additional predictors in the above described within-patient factor models, estimated separately for each CAFPA. Cross-level interactions between the within-patient condition variable and measurements tested whether the difference between the expert and the statistical model depended on the audiological measurements.

After performing the described statistical analyses, a post-survey interview with the experts was conducted. In a semi-structured discussion with all coauthors (from which two acted as experts), all results and links among the results were discussed, while especially focusing on the experts' perspective.

## Results

### Stability of experts' ratings and agreement between experts

Prior to assessing the agreement between statistical CAFPA predictions vs. experts' evaluations, the reliability of experts' ratings needs to be quantified. Table 1 provides a comprehensive summary of these reliability analyses for the 10 CAFPAs (displayed as columns). Within-expert agreements were very high as indicated by the ICC values close to 1. The ICCs expressing very high stability within Expert 2, who rated the CAFPAs many times repeatedly, are all above 0.90, with a very narrow CI. Thus, learning effects during the first round of ratings were adjusted by multiple repetitions in this case. The ICCs indicating stability within Expert 1 are somewhat lower, but satisfactory (all above 0.80), except for the CA1. However, CA1 was the CAFPA to be rated first, and the 15 patients used for stability estimates were presented as the first cases to the expert and repeated later. Thus, the low ICC of this first CAFPA can be explained by the fact that the expert had to familiarize himself with the task at the beginning of the survey. This was probably the case for the second expert as well; however, by analyzing “12 repetitions in that case,” the agreements were adjusted, and one run of ratings will not have such a substantial effect on the agreement estimates across 12 columns of 15 patients' ratings.

**Table 1:** Agreement between experts and stability of experts' ratings.

	**E1–E2 (agreement;** ***N*** = **15; rated first time by both experts)**		**E1–E1 (stability;** ***N*** = **15; rated 2 times)**		**E2–E2 (stability;** ***N*** = **15; rated 12 times)**	
**CAFPAs**	**ICC [CI]**	***p*-Value**	**ICC [CI]**	***p*-Value**	**ICC [CI]**	***p*-Value**
CA1	0.90 [0.72; 0.97]	0.00	**0.49** [−0.53; 0.83]	0.11	0.99 [0.99; 1.00]	0.00
CA2	0.96 [0.87; 0.98]	0.00	0.97 [0.92; 0.99]	0.00	0.99 [0.98; 1.00]	0.00
CA3	0.95 [0.86; 0.98]	0.00	0.99 [0.97; 1.00]	0.00	0.99 [0.98; 1.00]	0.00
CA4	0.92 [0.75; 0.97]	0.00	0.84 [0.53; 0.95]	0.00	0.98 [0.96; 0.99]	0.00
**CU1**	**0.52** [−0.43; 0.84]	0.09	0.89 [0.68; 0.96]	0.00	0.96 [0.92; 0.98]	0.00
CU2	0.94 [0.81; 0.98]	0.00	0.90 [0.71; 0.97]	0.00	0.98 [0.96; 0.99]	0.00
**CB**	singular	0.00	0.85 [0.54; 0.95]	0.00	0.92 [0.84; 0.97]	0.00
**CN**	**0.00** [−1.98; 0.66]	0.00	0.82 [0.47; 0.94]	0.00	0.96 [0.91; 0.98]	0.00
**CC**	**0.71** [0.15; 0.90]	0.01	0.96 [0.88; 0.99]	0.00	0.94 [0.88; 0.98]	0.00
CE	0.86 [0.58; 0.95]	0.00	0.97 [0.91; 0.99]	0.00	0.96 [0.92; 0.98]	0.00

CA1–CA4, hearing threshold-related CAFPAs; CU1–CU2, Suprathreshold-deficits related CAFPAs; CB, binaural hearing; CN, neural processing; CC, cognitive components of hearing; CE, socio-economic status; E1, Expert 1 who rated 15 patient cases two times; E2, Expert 2 who rated 15 patient cases 12 times; ICC, intra-class correlation; CI, confidence interval.

Bold numbers indicate estimated agreements with a lower than acceptable effect size.

Experts 1 and 2 were in high agreement with respect to all but three CAFPAs (refer to the first column of Table 1). The outlier CAFPAs were CU1, CB, and CN. In the case of CB and CN, the two experts did not agree with each other at all, such that the model returned a hint toward singularity. By exploring the distribution of the CB estimates within Expert 1 and Expert 2, it became obvious that the first expert evaluated all 15 patient cases used for reliability estimates with an approximately zero CB value and a very narrow value range slightly above zero in the case of CN. This was not the case for Expert 2 who used a somewhat broader but also restricted value range for these two CAFPAs. A post-survey interview with both experts provided further insights into the experts' reasoning on these patient cases with respect to CB and CN. These qualitative reports are outlined below in the discussion section and used for interpreting the quantitative findings summarized in Table 1. Overall, we can conclude that, for most of the CAFPAs, the experts' evaluations were reliable in terms of stability within experts and agreement of two different experts with different experience backgrounds.

### Relative agreement between CAFPAs predicted by statistical models vs. experts (RQ 1)

**Figure 3:**
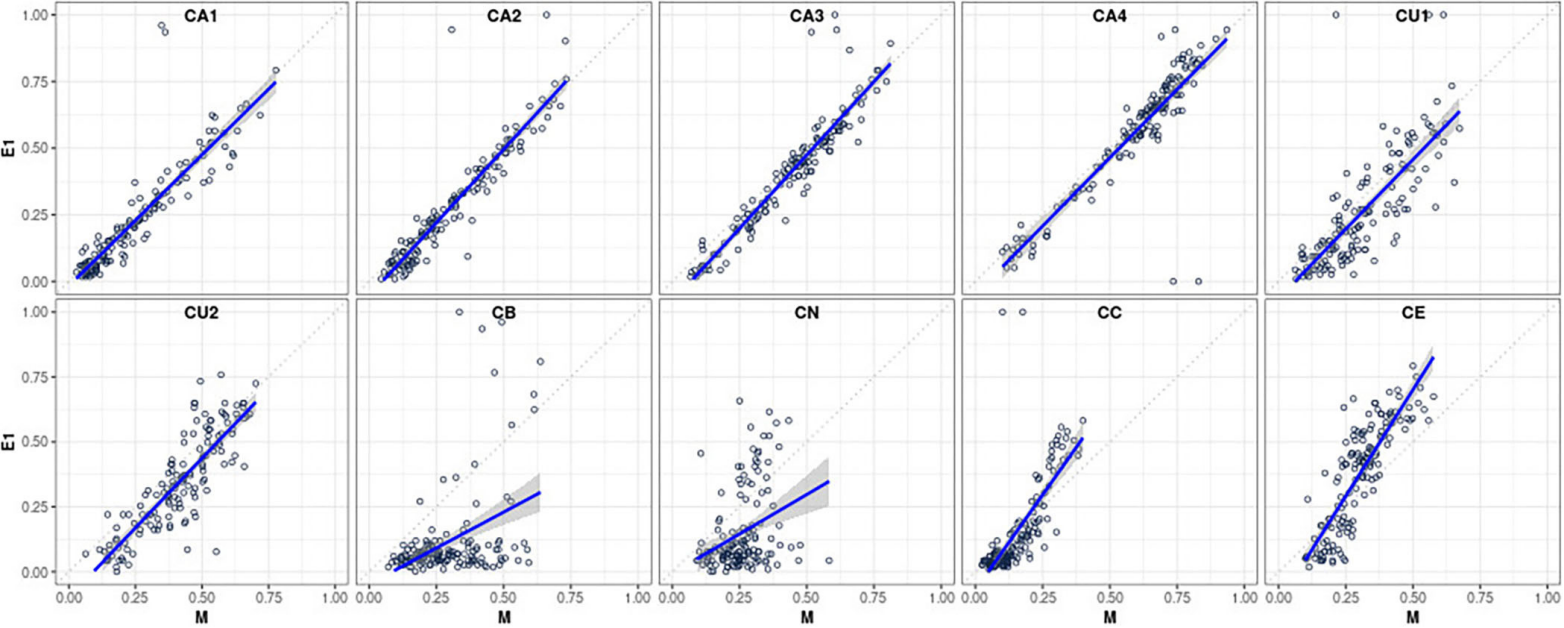
Scatterplots visualizing the relative agreement between statistically predicted CAFPAs and the expert's opinion (for Expert 1, *N* = 150 patients; corresponding to the second column of Table 2).

Table 2 provides a comprehensive summary of the ICC estimates indicating an agreement between the statistically predicted CAFPAs and the two experts based on 15 cases rated by all. The second column of the table indicates an agreement of CAFPA predictions between the statistical model and Expert 1 on the basis of 150 patients. These relative agreements between the models and Expert 1 are also displayed as scatterplots in Figure 3, separately for each CAFPA. The table and the scatterplots clearly reveal high agreement rates of experts with the statistically predicted CAFPAs, except for CB and CN. We can thus conclude that 8 out of 10 CAFPAs are valid and can be readily used in a CDSS for audiological decision-making. Reasons for the low validity of the statistically predicted CB and CN, as well as potential measures for improving the prediction of these two CAFPAs in the future, are discussed below.

**Table 2:** Relative agreement between statistically predicted CAFPAs and experts' opinion.

	**M-E1-E2**		**M-E1**	
**CAFPAs**	**ICC [CI]**	***p*-Value**	**ICC [CI]**	***p*-Value**
CA1	0.94 [0.85; 0.98]	0.00	0.94 [0.92; 0.96]	0.00
CA2	0.98 [0.94; 0.99]	0.00	0.96 [0.94; 0.97]	0.00
CA3	0.97 [0.93; 0.99]	0.00	0.96 [0.95; 0.97]	0.00
CA4	0.94 [0.87; 0.98]	0.00	0.94 [0.91; 0.95]	0.00
CU1	**0.73** [0.36; 0.90]	0.00	0.86 [0.80; 0.90]	0.00
CU2	0.94 [0.86; 0.98]	0.00	0.90 [0.86; 0.93]	0.00
**CB**	**0.63** [0.13; 0.87]	0.01	**0.56** [0.39; 0.68]	0.00
**CN**	**0.39** [−0.43; 0.78]	0.13	**0.43** [0.21; 0.59]	0.00
CC	0.88 [0.72; 0.96]	0.00	**0.75** [0.65; 0.82]	0.00
CE	0.91 [0.79; 0.97]	0.00	0.82 [0.75; 0.87]	0.00

CA1–CA4, hearing threshold-related CAFPAs; CU1–CU2, Suprathreshold-deficits related CAFPAs; CB, binaural hearing; CN, neural processing; CC, cognitive components of hearing; CE, socio-economic status; M, model = statistical model-predicted CAFPA, refer to Saak et al. (23); E1, Expert 1 who rated 15 patient cases two times and in total 150 different patients (used in second column M-E1); E2, Expert 2 who rated 15 patient cases 12 times; ICC, intra-class correlation; CI: confidence interval.

Bold numbers indicate estimated agreements with a lower than acceptable effect size.

### Absolute agreement between CAFPAs predicted by statistical models vs. experts (RQ 1)

We next investigated the absolute agreement between CAFPAs predicted by statistical models vs. experts. Despite proximal rank order equivalence of patients between experts and statistical decisions on the CAFPAs, the question remains whether, on average, across patients, experts, and the models agree. Table 3 provides a numeric summary of the results (see above for explanations of the modeling approach). As indicated by the first column of the table (β-weights), all but two differences were negative. This means that the CAFPAs CA1–CA4, CU1–CU2, CB, and CN were on average corrected across patients to lower values by Expert 1 as compared with the predictions of statistical models. On a scale between 0 and 100 (rescaled CAFPAs to range between 0 to 100, instead of 0 to 1), these negative differences ranged between 2.09 and 17.79 scale point units. Thus, most of the average differences between the expert's vs. the statistical models' CAFPA estimates were very small but significant. Larger deviations only occurred for CB and CN, for which statistical predictions turned out to be currently still insufficiently valid in terms of relative agreements as well. The cognitive processing and socio-economic CAFPAs (CC and CE) were rated on average across patients slightly higher by the expert as compared with the statistical models. However, the difference was not significant for CC.

**Table 3:** Main effect of evaluator in the linear mixed effects regression (LMER) models with evaluators (M and E1) nested within patients.

**CAFPAs**	**β (SE)**	**CI**	***p*-Value**
CA1	−2.09 (0.70)	−3.48; −0.71	0.00
CA2	−2.33 (0.64)	−3.60; −1.06	0.00
CA3	−3.20 (0.64)	−4.46; −1.94	0.00
CA4	−3.37 (0.85)	−5.05; −1.68	0.00
CU1	−5.14 (1.07)	−7.24; −3.04	0.00
CU2	−6.95 (0.83)	−8.60; −5.31	0.00
CB	−17.79 (1.43)	−20.61; −14.98	0.00
CN	−10.71 (1.24)	−13.61; −8.27	0.00
CC	0.27 (1.04)	−1.79; 2.33	0.79
CE	7.21 (1.05)	5.15; 9.27	0.00

CA1–CA4, hearing threshold-related CAFPAs; CU1–CU2, Suprathreshold-deficits related CAFPAs; CB, binaural hearing; CN, neural processing; CC, cognitive components of hearing; CE, socio-economic status.

Evaluator was dummy coded with 0 = machine learning model, 1 = expert (1). N_patients_ = 150. β: regression weight (fixed effect) of CAFPAs depending on the within-patient factor (machine learning model vs. expert); it indicates the difference between experts' ratings across patients on average as compared with the statistical model; SE, standard error of the regression weight estimate; CI, confidence interval.

### On the dependency of the disagreement between statistical models and the expert from patients' characteristics (RQ 2)

Given that expert and statistical predictions slightly but significantly differed on average, we explored whether patient characteristics (their audiological measurements) explain these differences. The modeling approach has been outlined above and the results are summarized in Table 4. For better readability, only significant effects are provided in the table. However, note that all listed interactions were estimated as explained above and in the note of the table.

**Table 4:** β-weights (of the cross-level interaction) indicating whether the difference between the expert and statistical model depends on the patients' audiological measures.

	Δ_**CA1**_		Δ_**CA2**_		Δ_**CA3**_		Δ_**CA4**_		Δ_**CU1**_		Δ_**CU2**_		Δ_**CB**_		Δ_**CN**_		Δ_**CC**_		Δ_**CE**_	
**Predictors**	**β**	***p*-Value**	**β**	***p*-Value**	**β**	***p*-Value**	**β**	***p*-Value**	**β**	***p*-Value**	**β**	***p*-Value**	**β**	***p*-Value**	**β**	***p*-Value**	**β**	***p*-Value**	**β**	***p*-Value**
Age																				
Sex			**3.24**	0.00					**6.15**	0.02									**−4.31**	0.02
PTA	**0.10**	0.01	**0.17**	0.00	**0.17**	0.00									**−0.42**	0.00				
SES																			**−2.70**	0.00
GOESA									**2.59**	0.00	**1.75**	0.00	**−2.03**	0.00	**4.18**	0.00				
WST																				
DemTect															**1.05**	0.03	**−2.10**	0.00		
Tinnitus*_*right*_*			**−4.73**	0.00																
Tinnitus*_*left*_*																				
ACALOS*_1.5*L*2.5_*	**−0.10**	0.00											**−0.21**	0.02						
ACALOS*_1.5*L*50_*			**0.11**	0.00					**0.22**	0.04					**−0.46**	0.00				
ACALOS*_4*L*2.5_*																				

Note that only significant results have been listed and an empty cell in the table indicates a null effect. Shaded rows or columns indicate that no significant results were obtained at all for the respective predictor or CAFPA.

CA1–CA4, hearing threshold-related CAFPAs; CU1–CU2, Suprathreshold-deficits related CAFPAs; CB, binaural hearing; CN, neural processing; CC, cognitive components of hearing; CE, socio-economic status.

Δ indicates the difference between the expert and the statistical models. p-values indicate the probability of observing the respective prediction of the difference, or more extreme ones, assuming the null hypothesis of no difference is true. The coefficient estimates originate from 10 different models, one model for each CAFPA. All predictors listed in the table were simultaneously included in the model, along with their interaction with the within-patient condition variable (model = 0; expert = 1). Thus, β-weights indicate cross-level interaction effects (within-patient condition variable and between-patient predictors as listed in the first column of the table).

The difference for CA4 does not depend on any patient characteristics, and for none of the CAFPAs, the difference between the expert and the model was associated with the age of the patients. In the post-survey interview (see also discussion below), experts also confirmed not to have considered the age when concluding about any of the CAFPAs. The difference between the statistical model and expert evaluation of the socio-economic CAFPA depended on the biological sex of the patients, which is plausible, given sex differences in status evaluations in society in general. Patient differences in pure tone average (PTA) explained the difference between the expert and the model on CA1-CA3. PTA also explained differences in the neural processing CAFPA; however, in general, the results of this CAFPA need to be interpreted with caution. The speech recognition in noise performance (see above GOESA) was relevant for the observed differences on CU1–CU2, CB, and CN. These results were also discussed with the experts in the post-survey interview and were in line with the experts' reports with respect to which measurements they considered when intending to correct the displayed model's estimated value for a given CAFPA. Finally, Adaptive Categorical Loudness Scaling (ACALOS) further contributed to accounting for the difference between the expert and the statistical model.

### Questionnaire about experts' approach and relationships between measurements and CAFPAs (RQ 4)

The general questionnaire part of the survey provided additional subjective information to be linked with the analysis outcomes. The answers (by Expert 1) about the expert validation approach revealed that the expert considered patient characteristics as a complete picture. In addition, specific links between measurements and CAFPAs were considered from both directions, that is, thinking about which measurement information was important for a certain CAFPA, as well as to which CAFPAs a certain measurement contributed. The exact choice and formulation of answers are provided in the Supplementary Table A1.

Related to that, the questions about associations between CAFPAs and a respective measurement provided more detailed information about the links indicated by the expert. The CAFPAs CA1–CA4 were clearly related to the audiogram; the cognitive CAFPA CC to the verbal intelligence test (WST) and to DemTect; and the socio-economic CAFPA CE to the SWI. In contrast, CU1–CU2 and CN were related to a combination of audiogram, ACALOS, GOESA, native language, and verbal intelligence test. The binaural CAFPA was not linked to any measurement, meaning that the expert found no information about this aspect in the patient characteristics. These links are plausible and comparable to the results of the statistical analyses as described above, as well as to the variable importance analysis by (23).

### CAFPA distributions for given audiological findings (RQ 3)

Finally, we investigated the differences between model-predicted and expert-validated CAFPAs sorted to audiological findings as estimated by the experts, for the purpose of performing a plausibility check in the applied context toward a CDSS. From the 150 patient cases evaluated by Expert 1, the combinations of four audiological findings were mainly chosen: normal hearing, high-frequency hearing loss, broadband hearing loss, and the combination of high-frequency and broadband hearing loss. Other findings were chosen very rarely (less than six).

**Figure 4:**
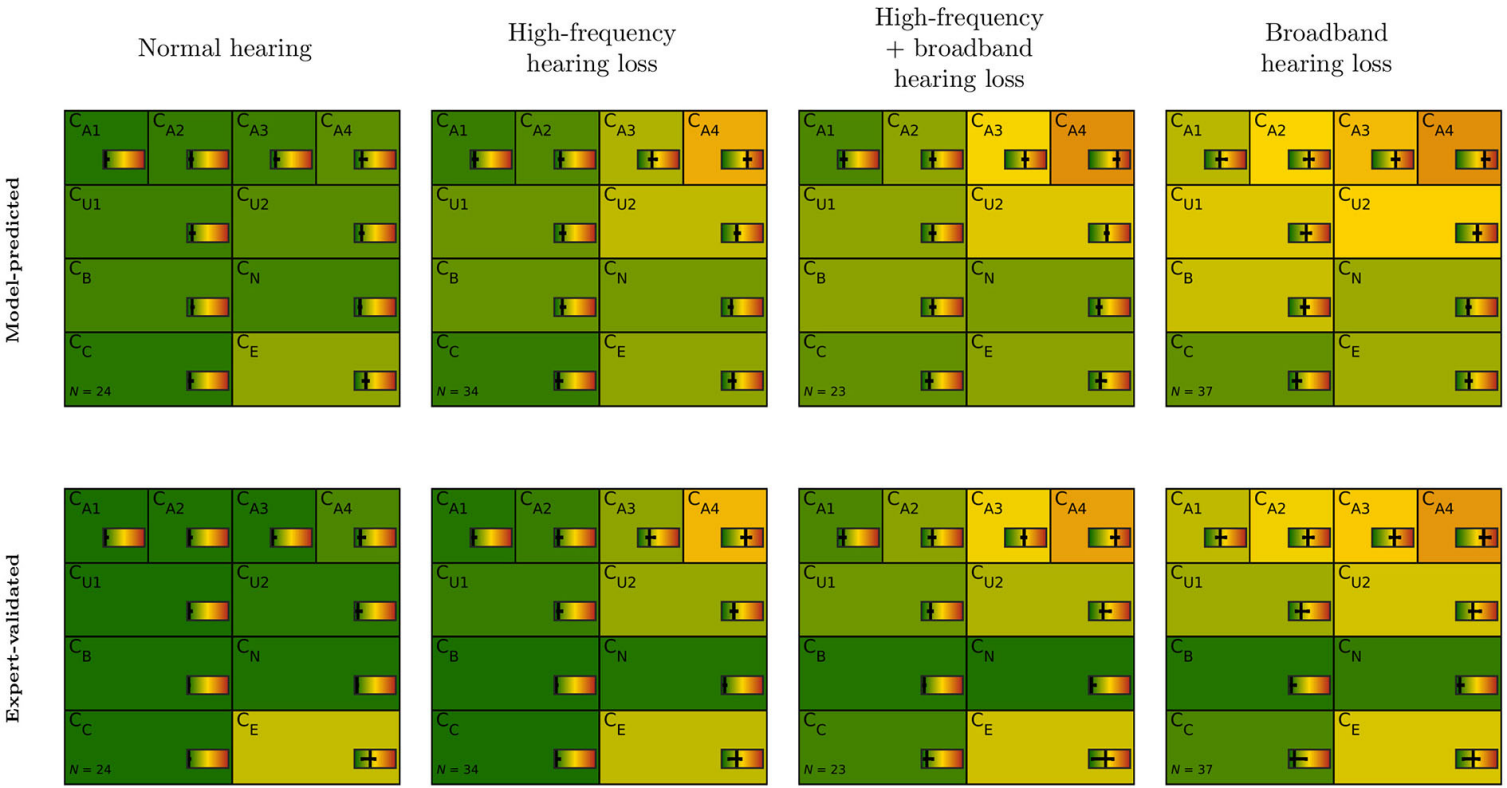
CAFPA patterns for the four most frequent audiological findings (columns) as indicated by Expert 1. Model-predicted (first row) and expert-validated CAFPAs (second row). The background color represents the median of the respective CAFPA for all patients associated to the respective audiological finding. The horizontal color bar includes the interquartile range in addition to the median.

Figure 4 depicts model-predicted and expert-validated CAFPAs for different audiological findings. Usually, only small differences are visible by comparing the median (background color) of model-predicted and expert-validated CAFPAs. Thus, the differences as described above comprise a small influence of CAFPAs as compared to the possible range and vary only a little across audiological findings. Interquartile ranges of CAFPAs within audiological findings are partly larger for expert-validated CAFPAs, showing that the expert found slightly more variability across patient cases than was covered by the prediction models. For CB (binaural) and CN (neural), the correction toward zero as described above influenced all audiological findings in the same way, resulting in median values close to zero and a very small interquartile range. A more detailed view on interquartile ranges along with distributions of the different CAFPAs is displayed in Supplementary Figure A3.

## Discussion

The present study aimed at an expert validation of model-predicted CAFPAs to be used as an intermediate layer in a CDSS for audiology. For this purpose, we performed an expert survey with two highly-experienced audiological experts and statistically analyzed differences between model-predicted and expert-validated CAFPAs, as well as associations of the observed differences with audiological measurements and patient characteristics.

### Expert validation of model-predicted CAFPAs

The main finding was that experts agreed on most model-predicted CAFPA values, except for the binaural CAFPA CB, and the neural CAFPA CN (RQ 1). For these, in a considerable number of patients, large corrections were proposed by experts. This finding was consistently revealed by different statistical analyses, i.e., the assessment of relative and absolute agreement between experts and prediction models, the questionnaire inquiring about the experts' validation approach and their understanding of the relationships between audiological measurements and the different CAFPAs, and the evaluation of CAFPAs aligned to expert-estimated audiological findings.

For all CAFPAs, except for CB and CN, experts proposed only small corrections on the model-predicted CAFPAs given the measurement data of a sample of patients. Therefore, we conclude that the model-based prediction of these CAFPAs is already well applicable to unlabeled patients. Slight potential for improvement can however be inferred based on the results obtained. The relative agreement between prediction models and both experts was high, except for the supra-threshold CAFPA CU1. The same was applied to the cognitive CAFPA CC when assessing the agreement between model-predicted CAFPAs and Expert 1. Consequently, the agreement among the two experts was rather narrow, but still acceptable for CU1 and CC.

Interestingly, the main evaluator effect (absolute agreement) assessed between prediction models and Expert 1 was significant for all CAFPAs, but not CC. That is, the cognitive CAFPA was on average across patients not corrected by the expert. This could be due to the fact that the range of available patient data is restricted especially in the case of CC where low CAFPA values represent typical functioning. According to the variable importance analyses by Saak et al. (23) and the experts' reports, the CC CAFPA was mainly estimated and concluded on the basis of the DemTect scores, which is a screening test for cognitive impairment. DemTect scores in the present sample, however, are rather in the typically functioning range.

Linear mixed effects regression models revealed that the small, but statistically significant evaluator effects, reflecting differences between the model-predicted and expert-validated CAFPAs, on all remaining seven CAFPAs followed mostly plausible associations with audiological measurements (RQ 2). For instance, analyses indicated that patients' GOESA scores were significantly associated with four CAFPAs, namely CU1, CU2, CB, and CN. This relationship is especially plausible for the supra-threshold CAFPAs, CU1, and CU2, as well as the neural CAFPA CN (see below). However, theoretically one would expect that the binaural CAFPA would not be associated with GOESA, which was measured in the S0N0 condition (speech and noise from the frontal direction), i.e., binaural processing should not be characterized by the given speech test outcome. Furthermore, these empirical relationships were in line with the experts' responses in the questionnaire where they were asked to indicate expected links between audiological measurements and the different CAFPAs. This procedure is similar to the variable importance analysis of Saak et al. (23), which illustrated the links between audiological measurements (features) and the CAFPAs by means of statistical associations learned from the labeled part of the dataset.

In contrast, for the binaural CAFPA CB and the neural CAFPA CN, the relative agreement between experts and the prediction model was limited. The absolute agreement analyses showed the largest differences between model-predicted and expert-validated CAFPAs, for these among all other CAFPAs as well (RQ 1). These findings can be interpreted in the light of all analyses conducted in the present study. The difference between the model-predicted vs. expert-validated binaural CAFPA CB was associated with patients' scores on GOESA and ACALOS, while the expert indicated in the questionnaire that none of the provided measurements allows for conclusions about this CAFPA. In a post-survey interview with both experts, the questionnaire statement was confirmed one more time. That is, according to both experts, the available measurements displayed in the expert survey and used for statistical predictions of CAFPAs do not provide sufficient information about binaural processing (RQ 2). This assessment is consistent with the literature (34–39). Both experts agreed in the joint interview that information from a localization task, as well as speech intelligibility measured in a spatial condition, would be needed for CB evaluation, whereas the displayed condition for GOESA was S0N0. However, Expert 1 also reported being able to gain an impression of the binaural hearing abilities of patients from the available data. A potential decision strategy would be as follows: One would adapt the CAFPA CB toward zero (green, normal) if no binaural problem was expected in the light of all other measurements provided. Therefore, in the case of CB, the absolute agreement and relationships with the audiological measurements need careful interpretation in line with these reports of the expert. Nevertheless, the revealed associations by the statistical analyses may also indicate experts' implicit assumptions about the measurements which are not explicated in their decision-making process.

The evaluator effects for the neural CAFPA CN were associated with several measurements, namely the audiogram (PTA), GOESA, DemTect, and ACALOS. Out of these, GOESA was most strongly associated with CN updates by the expert. These associations are mainly consistent with the questionnaire reports. However, in the post-survey interview, Expert 1 emphasized again his decision-making strategy and commented on the importance of these measurements for the assessment of the neural CAFPA CN. According to both experts, generally in clinical practice, the challenge persists with evaluating neural aspects of hearing loss. These can be characterized by certain measurements such as brainstem-evoked response audiometry or electrocochleography (31), but there is no common and established selection of measurement approaches, and the availability of such measures largely varies across patient cases. Therefore, experts' diagnostic decision-making process contains several steps. They reported to first consider the audiogram and a speech test in combination, and only if inconsistencies pop up, additional measurements, such as brainstem-evoked response audiometry or electrocochleography would be potentially suggested. This diagnostic rationale explains the approach explicated by Expert 1 on how he approached the validation task: CN for patients with consistent results among the audiogram and GOESA has been corrected toward zero. Thereby, the expert validation of CN relies on the partially explicated diagnostic rationale only, given that no additional information on neural sources of hearing loss was available in the studied patient database. These aspects need improvement toward a reliable CDSS algorithm in the domain of CN and also CB.

The audiological findings as estimated by the experts provided further opportunities to assess how decisive differences between model-predicted and expert-validated CAFPAs were for the final diagnostic outcome (RQ 3). The CAFPA patterns of patients sorted into distinct classes according to the experts' labels for audiological findings were consistent with those which were statistically derived by Saak et al. (23) when clustering unlabeled cases based on model-predicted CAFPAs. The most frequently occurring diagnostic findings (normal hearing, high-frequency hearing loss, broadband hearing loss, and a combination of high-frequency and broadband hearing loss) are approximately equally distributed. This is a consistency check, given that the patients for the current survey were chosen to equally represent the clusters of Saak et al. (23). By comparing the CAFPA distributions (median) of model-predicted and expert-validated CAFPAs, we found in general no noticeable changes in the CAFPA patterns for all CAFPAs except for CB and CN. That is, the above-discussed approach of the experts (correcting these CAFPAs toward zero if no inconsistencies in the data were present) had a similar impact on all audiological findings. This is plausible given that the employed categories of audiological findings [as introduced in Ref. (20)] mainly relate to audibility, and most of the patients did not show extreme findings with regard to binaural hearing or neural aspects of hearing loss. This is in general a property of the database which contains mainly mild-to-moderate hearing impairment collected in a pre-clinical context for the purpose of hearing aid fitting.

In summary, the performed expert validation and corresponding statistical analyses revealed that the CAFPA prediction models as trained by Saak et al. (23) are applicable to unlabeled patient cases. For all CAFPAs except for CB and CN, the expert-validated CAFPAs as well as the audiological findings collected in this study can be additionally used for further training of the prediction models.

For CB and CN, the current prediction models need improvement by considering additional measurements. In these cases, with the measurement data at hand, experts indicated the respective CAFPAs to be normal if no inconsistencies were observed in the data. They both concluded that additional information was necessary to evaluate CB and CN. It is thus plausible that the expert's diagnostic decision-making approach for these two CAFPAs is not reflected by the models that learn from the multivariate association pattern of the audiological test battery taken as input and are by design not able to apply If-Then rules in a similar way as experts do. However, the current predictions are still useful as a starting point or the first best guess for CB and CN. Future models need to be trained on additional information for these two CAFPAs on a potentially more comprehensive clinical sample.

### On the importance of experts' qualitative reports on their decision-making approach to improving statistical predictions

The present study clearly demonstrated the importance of combining expert knowledge and statistical learning in the design of a CDSS for audiology. The expert validation and corresponding statistical analyses to investigate agreement between model-predicted and expert-derived CAFPAs provided important insights into the current properties and the necessary future improvement of the CDSS proposed by Buhl (22) and the prediction models of CAFPAs (23). Furthermore, the collected qualitative data on the experts' decision-making process are highly valuable to complement statistical conclusions.

Questionnaire reports revealed that the experts were confident in evaluating model-predicted CAFPAs and combining these statistical proposals with their views on the respective audiological findings (RQ 4). First, this conclusion is supported by plausible expert-validated CAFPAs, which are consistent with the indicated links between measurements and CAFPAs by experts in the questionnaire. Second, the questionnaire also assessed the experts' approach to the task. These data confirmed that Expert 1 was comfortable with the task of making diagnostic decisions on the basis of proposed solutions achieved by statistical predictions. The concept of CAFPAs was also valued by the expert. In summary, the expert concluded a case based on an overall impression of the patient in terms of measurements as well as CAFPAs and additionally reflected upon the respective links between these two information sources. As a limitation, it should be however mentioned that only two audiological experts were involved in this study, and future studies will need to validate a designed CDSS on additional experts with different levels of experience. The two experts involved in this study are highly experienced and provided valuable insights and opinions in a post-survey interview. Their suggestions are consistent with literature, e.g., regarding their reported limitations, such as insufficient available measurements for CB and CN hitherto considered for deriving these CAFPAs. Future studies with more experts with varying levels of experience could assess how the approach to correcting CAFPAs and associations between measurements and CAFPAs implied by the experts' opinion depend on experts' experience. Also, it could be investigated which level of experience is required to perform the expert validation task accurately. It will be crucial that only experts are included who are sufficiently familiar with the typical audiological diagnostic process and are well acquainted with the CAFPA concept. Their knowledge may be structured differently depending on the experience. Potentially, experts have more implicit links between different aspects of the audiological diagnostic process given higher levels of experience.

The current expert validation was highly informative for the successful implementation of CAFPAs for designing a CDSS for audiology (RQ 4): (1) The model-predicted CAFPAs were validated here by experts, (2) the expert-validation data were statistically analyzed, and (3) qualitative questionnaire and post-survey interview reports of the experts provided a consistency check and additional insights on the experts' decision-making process (9, 10, 13), as discussed above. Thereby, experts' opinions collected here assure the use of CAFPAs in the context of CDSS (2). It should be mentioned that the present expert survey was closely related to the expert survey procedure of Buhl et al. (20). This ensures comparability of the obtained experts' labels and diagnostic conclusions. However, there was a crucial difference. The present study employed an expert validation of model-predicted CAFPAs for previously unlabeled cases instead of simple labeling of CAFPAs. This has the advantage to provide information on how experts accept diagnostic conclusions suggested by a data-driven diagnostic approach.

In summary, the present study contributed to linking expert knowledge and machine learning toward the development of a CDSS for audiology. This link needs to be interpretable. Interpretability was assured in several regards in the current CDSS (22) as well as in the analysis applied in this study. First, the CAFPAs themselves act as an interpretable intermediate layer of a CDSS (19). Second, the variable importance assessments in Saak et al. (23) provided a basis for interpretability of the statistical learning models and allowed insights into the underlying measurements for the different CAFPAs. Third, in the present study, by means of linear mixed effect models, we investigated how differences between model-predicted and expert-validated CAFPAs depend on audiological measurements of the patients. Thereby, we could learn about the experts' implicit approach and interpretation of the CAFPA concept. Although the current version of the CDSS based on CAFPAs was built upon only one audiological database, the proposed methodological approach is generalizable to further data of a similar structure.

### Toward future application in the clinical decision-support system and outlook

The outcomes of the present study provide insights into how the CDSS of Buhl (22) could be further improved toward applicability for new patients. For all CAFPAs except for the binaural CAFPA CB and the neural CAFPA CN, the prediction models of Saak et al. (23) can be improved by including the expert-validated CAFPAs as additional labels in the training process and thereby taking the proposed corrections of the two experts involved in this study into account. In the future, this could be done even more efficiently, for example, by using a procedure as described by Baur et al. (13). There, an iterative data annotation approach has been suggested. First, a machine learning algorithm is trained based on a number of available labeled data points, and then, expert labeling is included iteratively by presenting experts with those respective data points that show the most uncertain labels.

For CB and CN, the prediction models of Saak et al. (23) are not yet accurate enough in their current version for use in a CDSS. The automatic prediction of the binaural CAFPA should be included in the future as soon as a database with appropriate audiological measurements is available. The neural CAFPA will require even more research to be included in the decision-support system. This is because the diagnostic process for neural aspects of hearing loss is not well-defined by domain experts, not even with respect to the choice of necessary measurements for a straightforward diagnostic. More specifically, including CN, further discussions with clinicians from different sites are needed to learn more about which measurements are employed for which patients in the clinical practice. Second, appropriate datasets need to be accessed that contain consistent measurement outcomes across patients. This step may include existing datasets, but it may also be necessary to collect structured data for a new group of patients. Third, if data are available, expert labels for CAFPAs can be collected, and/or CAFPAs can be predicted, and a subsequent expert validation be performed (see below for a discussion about expert validation for including additional databases).

The integration of additional databases including more balanced and more severe patient cases is required not only to back up the CDSS with a larger number of patients but also to cover the whole range of potential audiological findings and treatment recommendations. Therefore, the CAFPAs provide great potential, as they are defined as a measurement-independent representation of audiological knowledge. The applied expert-validation approach can be used in the future to validate CAFPAs that were predicted on the basis of different audiological measurements and variable amounts of information available for different patients. This is relevant because clinical practice is characterized by heterogeneity in data availability for different patient cases. In this respect, the expert validation approach could be included in two ways in a hybrid ML-based CDSS combining machine learning and expert knowledge. On the one hand, as explained above, expert validation can be used to derive corrected CAFPAs for additional measurement information in a to-be-connected database. Thereby, it could also be beneficial if the specialization of a respective expert corresponds to the new measurements contained in a dataset. On the other hand, the expert validation could be used on the basis of single patients during the operation of the CDSS in clinical practice, i.e., if the uncertainty of the predicted CAFPAs (or classified audiological finding or treatment recommendation) exceeds a certain threshold, the system would ask for an expert validation of CAFPAs for the respective patient [related to the approach of Ref. (13)]. In this case, either the current physician could be asked to expert-validate the CAFPAs, or the CDSS would not continue for the current patient, but the patient's data and CAFPAs would be stored to later perform (offline) expert validation on such stored cases.

In contrast to knowledge- or rule-based CDSS (40), expert knowledge would not explicitly be modeled to be incorporated in an ML-based CDSS. Instead, expert knowledge is implicitly incorporated into the CDSS, as it is included in the data (labels for CAFPAs or diagnostic cases) and the relationships between different layers of the CDSS (audiological measures, CAFPAs, and diagnostic cases) are derived from data (supervised ML). With expert validation as performed in this study, the data (CAFPAs) underlying these relationships can be optimized to best fit to experts' implicit understanding of the relationships.

Overall, the present study demonstrated not only the need, but also the potential to incorporate diverse information on expert knowledge in the development (and application) of a CDSS.

## Conclusion

The present study provided important insights into the advantages, limitations, and potential improvement of the current prediction of CAFPAs.

The performed expert validation and corresponding statistical analyses revealed that the current CAFPA prediction models are applicable to unlabeled patient cases. For all CAFPAs except for the binaural CAFPA CB and neural CAFPA CN, the experts' agreement with the model-predicted CAFPAs was high, and only small corrections were performed, which were associated with plausible underlying audiological measures by the linear mixed effect models. Therefore, the expert-validated CAFPAs can be employed as additional labels for further training of the respective CAFPAs ‘prediction models.

In contrast, large corrections were performed for the CAFPAs CB and CN. The expert's approach of correcting these CAFPAs toward zero if the overall impression of the patient was normal was revealed by the post-interview, along with the fact that appropriate measurement information was missing in the database. The current predictions are useful as a starting point or the first best guess for CB and CN, but future models need to be trained on additional information for these two CAFPAs on a potentially more comprehensive clinical sample.

Audiological findings were found to be consistent with previous expert labels on the same data set. Due to the definition of these categories mainly in threshold-related terms, the large corrections for CB and CN similarly affected all audiological findings.

In summary, the present study contributed to linking expert knowledge and machine learning toward the development of a CDSS for audiology. By means of linear mixed effect models, we investigated how differences between model-predicted and expert-validated CAFPAs depend on audiological measurements of the patients. Thereby, we could learn about the experts' implicit approach and interpretation of the CAFPA concept. Although the current version of the CDSS based on CAFPAs was built upon only one audiological database, the proposed methodological approach is generalizable to further data of a similar structure.

In the future, the expert validation approach could also be used to establish relationships with additional measurements included in different databases. If a prediction is performed on parts of a database, experts could be asked to validate and correct the predicted CAFPAs based on a larger choice of measurements presented within the expert validation survey.

## Data availability statement

The data analyzed in this study was obtained from Hörzentrum Oldenburg gGmbH, the following licenses/restrictions apply: According to the Data Usage Agreement of the authors, the datasets analyzed in this study can only be shared upon motivated request. Requests to access these datasets should be directed to MB, mareike.buhl@uni-oldenburg.de and AH, andrea.hildebrandt@uni-oldenburg.de. The analysis scripts can be found at Zenodo, https://zenodo.org/, https://doi.org/10.5281/zenodo.6817974.

## Ethics statement

Ethical review and approval was not required for the study on human participants in accordance with the local legislation and institutional requirements. Written informed consent from the patients/participants or patients/participants' legal guardian/next of kin was not required to participate in this study in accordance with the national legislation and the institutional requirements'.

## Author contributions

AH, MB, and GA contributed to the conception and design of the study. MB organized the database. GA implemented and conducted the expert survey. AR and UE participated as experts. GA, SS, MB, and AH contributed to the analysis of the results. MB and AH wrote the first draft of the manuscript. All authors discussed the results in the post-interview, contributed to manuscript revision, read, and approved the submitted version.

## Funding

This work was funded by the Deutsche Forschungsgemeinschaft (DFG, German Research Foundation) under Germany's Excellence Strategy – EXC 2177/1 – Project ID 390895286.

## Conflict of interest

The authors declare that the research was conducted in the absence of any commercial or financial relationships that could be construed as a potential conflict of interest.

## Publisher's note

All claims expressed in this article are solely those of the authors and do not necessarily represent those of their affiliated organizations, or those of the publisher, the editors and the reviewers. Any product that may be evaluated in this article, or claim that may be made by its manufacturer, is not guaranteed or endorsed by the publisher.
