# Supplementary material for: Expert validation of prediction models for a clinical decision-support system in audiology
Source: Front Neurol. 2022 Aug 23;13:960012. doi: 10.3389/fneur.2022.960012 (PMC9446152; doi:10.3389/fneur.2022.960012)
Supplement: Supplementary file 1 [file Data_Sheet_1.PDF]

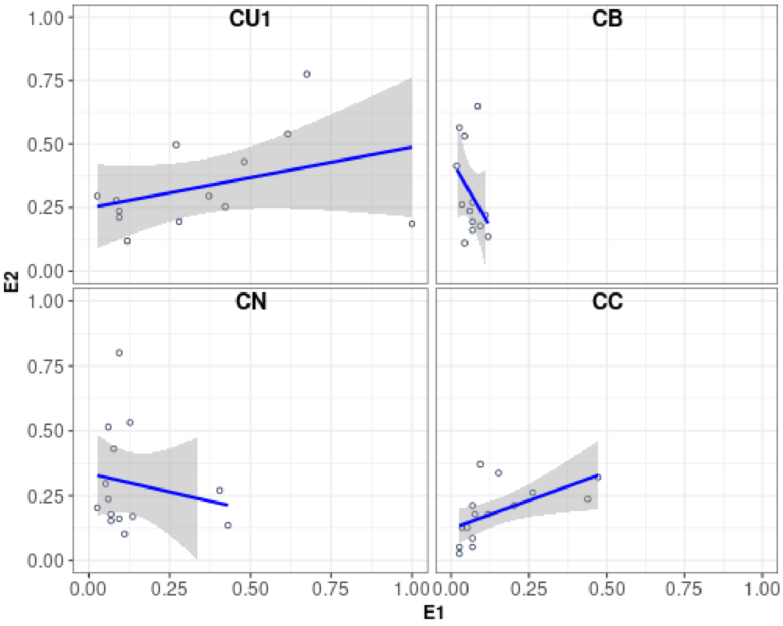


Figure A1. Scatterplots visualizing the agreement among experts (E1-E2) for CAFPAs with low agreement, for $N$ = 15 patients, rated for the first time by both experts (cf. Table 1, first column).

Table A1. Questionnaire results for expert validation approach (Expert 1). All presented statements (translated) are displayed, along with the chosen statements in first and second run of this question.

| Statement | Run 1 | Run 2 |
|---|---|---|
| I first obtained an overview of the patient based on the measurement results. | | x |
| I first looked at CAFPAs because they represent the target quantity. | x | |
| I compared the overall impression of the measurement results and the overall impression of the represented CAFPAs (start values). | | |
| I adapted those CAFPAs that didn't match my impression of the patient. | | x |
| For the adaptation of each CAFPA, I specifically considered certain information. | | x |
| For each CAFPA, I first shifted the slider to the start position and then estimated if the value is correct. | | |
| For each CAFPA, I directly shifted the slider to the final position, without shifting it to the start position. | | x |
| I considered all CAFPAs together as overall impression of the patient. | | |
| I considered each CAFPA separately and performed the validation by considering which measurement information contributes to the respective CAFPA. | | |
| I considered each CAFPA separately and performed the validation based on my overall impression of the patient. | x | |
| For each measurement, I thought about to which CAFPAs the respective information contributes. | | x |

Table A2. Questionnaire results for links between measurements and CAFPAs (Expert 1). In the survey, each of the displayed rows (measurements) was shown as separate question (translated): *For measurement X: To which CAFPAs does this information contribute? If the measurement does not contribute to any CAFPA: Why not?*

| | CAFPAs | | | | | | | | | | Reason | | |
|---|---|---|---|---|---|---|---|---|---|---|---|---|---|
| | $C_{A1}$ | $C_{A2}$ | $C_{A3}$ | $C_{A4}$ | $C_{U1}$ | $C_{U2}$ | $C_B$ | $C_N$ | $C_C$ | $C_E$ | Measure-ment not known | Not important for patient characteri-zation | Not coded/ represented in CAFPAs |
| Run 1 | | | | | | | | | | | | | |
| Audiogram (air conduction) | X | X | X | X | X | X | | X | | | | | |
| Audiogram (bone conduction) | X | X | X | X | X | X | | X | | | | | |
| ACALOS | | | | | | | | | | | | | |
| GOESA | | | | | | | | | | | | | |
| Language | | | | | | | | | | | | | |
| Age | | | | | | | | | | | | X | |
| Gender | | | | | | | | | | | | X | |
| Tinnitus | | | | | | | | | | | | | X |
| Hearing problems (quiet) | | | | | X | X | | X | | | | | |
| Hearing problems (noise) | | | | | X | X | | X | | | | | |
| WST | | | | | | | | | X | X | | | |
| SWI | | | | | | | | | X | X | | | |
| DemTect | | | | | | | | | X | | | | |
| Run 2 | | | | | | | | | | | | | |
| Audiogram (air conduction) | X | X | X | X | X | X | | | | | | | |
| Audiogram (bone conduction) | | | | | X | X | | | | | | | |
| ACALOS | | | | | X | X | | | | | | | |
| GOESA | | | | | X | X | | | | | | | |
| Language | | | | | X | X | | X | | | | | |
| Age | | | | | | | | | | | | X | |
| Gender | | | | | | | | | | | | X | |
| Tinnitus | | | | | | | | | | | | X | |
| Hearing problems (quiet) | | | | | | | | | | | | X | |
| Hearing problems (noise) | | | | | | | | | | | | X | |
| WST | | | | | X | X | | | | | | | |
| SWI | | | | | | | | | | X | | | |
| DemTect | | | | | | | | X | | | | | |

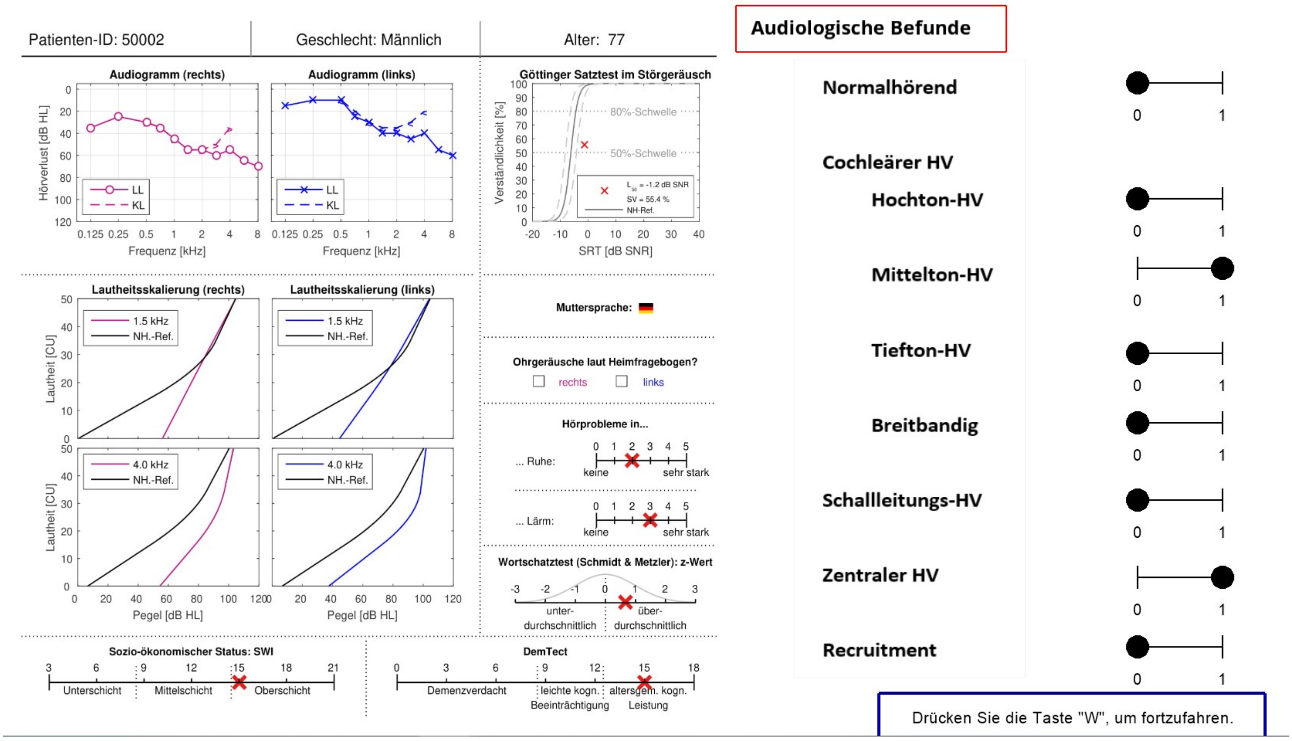


Figure A2. Patient data and evaluation sheet for audiological findings as implemented in the electronic version of the expert survey. Patient cases were displayed one at a time.

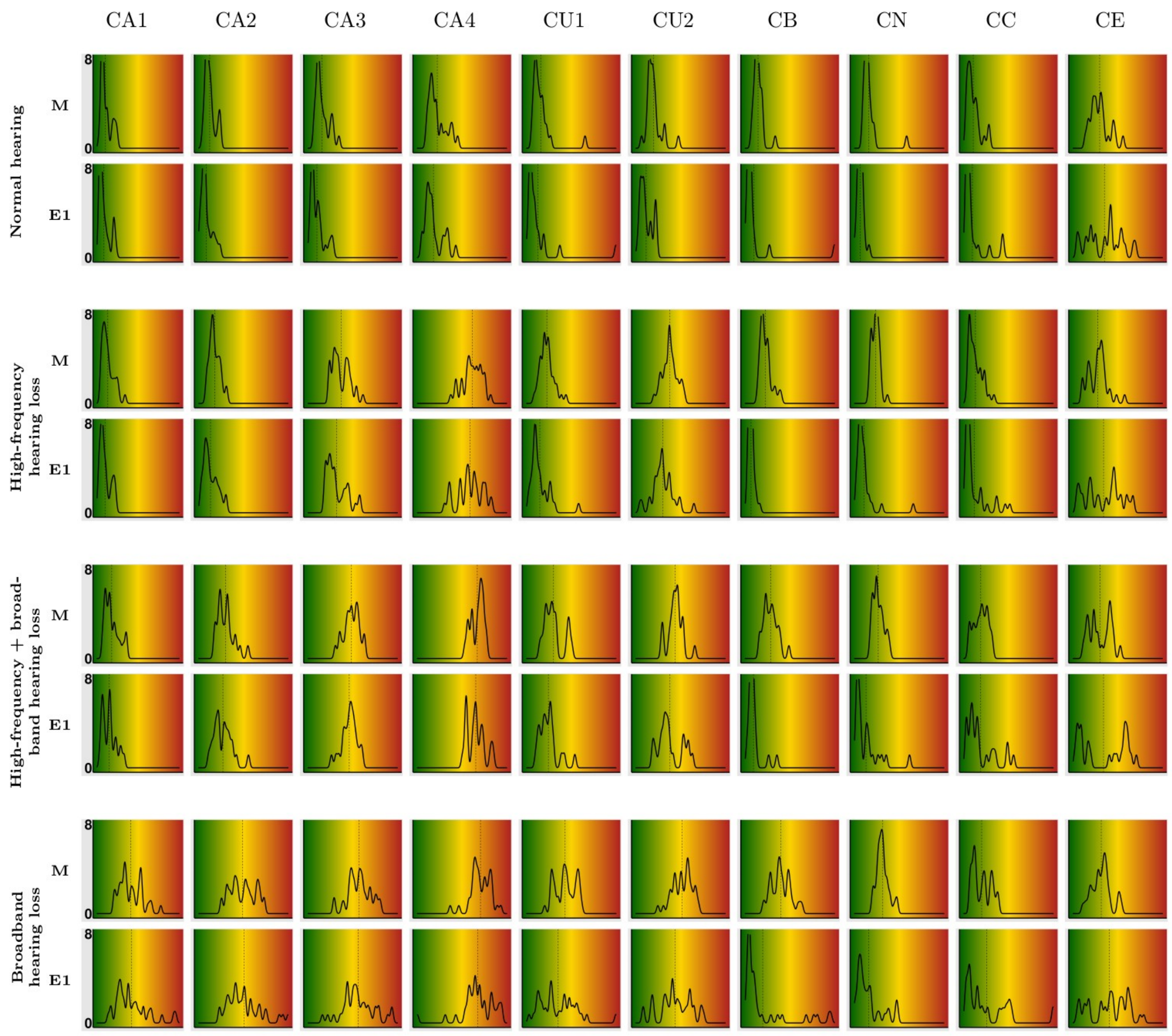


Figure A3. Absolute frequency density plots (bandwidth = 0.015) for all CAFPAs (columns). For the four most frequent audiological findings, the model-predicted and expert-validated CAFPAs are compared row-wise. The vertical dashed line represent the median of the distribution, the background represents the corresponding CAFPA value range from green (0) to red (1).
